# Metallo-Glycodendrimeric Materials against Enterotoxigenic *Escherichia coli*

**DOI:** 10.3390/microorganisms12050966

**Published:** 2024-05-11

**Authors:** Aly El Riz, Armelle Tchoumi Neree, Leila Mousavifar, René Roy, Younes Chorfi, Mircea Alexandru Mateescu

**Affiliations:** 1 Department of Chemistry, Université du Québec à Montréal, Succ. Centre-Ville, P.O. Box 8888, Montréal, QC H3C 3P8, Canada; el_riz.aly@courrier.uqam.ca (A.E.R.); leilyanmousavifar@gmail.com (L.M.); roy.rene@uqam.ca (R.R.); 2 Department of Veterinary Biomedicine Sciences, Faculty of Veterinary Medicine, Université de Montréal, St-Hyacinthe, QC J2S 2M2, Canada; armelle.tchoumi.neree@umontreal.ca (A.T.N.); younes.chorfi@umontreal.ca (Y.C.); 3 Centre de recherche en infectiologie porcine et avicole (CRIPA), Université de Montréal, St-Hyacinthe, QC J2S 2M2, Canada

**Keywords:** bactericidal activity, enterotoxigenic *Escherichia coli* fimbriae 4 (ETEC:F4), molecular insights, DNA release, RNA release, glycodendrimers, copper-loaded glycodendrimers (D:Cu), silver-loaded glycodendrimers (D:Ag)

## Abstract

Conjugation of carbohydrates to nanomaterials has been extensively studied and recognized as an alternative in the biomedical field. Dendrimers synthesized with mannose at the end group and with entrapped zero-valent copper/silver could be a potential candidate against bacterial proliferation. This study is aimed at investigating the bactericidal activity of metal-glycodendrimers. The Cu(I)-catalyzed azide–alkyne cycloaddition (CuAAC) reaction was used to synthesize a new mannosylated dendrimer containing 12 mannopyranoside residues in the periphery. The enterotoxigenic *Escherichia coli* fimbriae 4 (ETEC:F4) viability, measured at 600 nm, showed the half-inhibitory concentration (IC_50_) of metal-free glycodendrimers (D), copper-loaded glycodendrimers (D:Cu) and silver-loaded glycodendrimers (D:Ag) closed to 4.5 × 10^1^, 3.5 × 10^1^ and to 1.0 × 10^−2^ µg/mL, respectively, and minimum inhibitory concentration (MIC) of D, D:Cu and D:Ag of 2.0, 1.5 and 1.0 × 10^−4^ µg/mL, respectively. The release of bacteria contents onto broth and the inhibition of ETEC:F4 biofilm formation increased with the number of metallo-glycodendrimer materials, with a special interest in silver-containing nanomaterial, which had the highest activity, suggesting that glycodendrimer-based materials interfered with bacteria-bacteria or bacteria–polystyrene interactions, with bacteria metabolism and can disrupt bacteria cell walls. Our findings identify metal–mannose-dendrimers as potent bactericidal agents and emphasize the effect of entrapped zero-valent metal against ETEC:F4.

## 1. Introduction

Enterotoxigenic *Escherichia coli* (ETEC) bacteria are the main cause of enteric swine diseases [1] and were intensively studied in porcine post-weaning diarrhea characterized by growth retardation of piglets, dysbiosis, hemorrhagic diarrhea, pathogenic infections due to hyperpermeability of enterocytes or sudden death, causing economic upheaval in pig production [2,3,4]. The ETEC bacteria were found in pig foods, slaughterhouses, or contaminated instruments in contact with the animals. The prophylactic strategies to prevent bacterial infections are based on cleaning and sterilizing the swine environment. Fecal contamination is the main vehicle for zoonotic pathogens [5,6,7,8]. Several pathogenic bacteria, including ETEC, found in livestock can contaminate water, soil and farm environments and cause disease in humans and pets [9,10]. ETEC are characterized by the ability to produce two types of virulent factors: adhesins promoting binding to specific enterocyte receptors for intestinal colonization and enterotoxins responsible for fluid secretion [11]. During the disease, the proliferation of ETEC induces a disequilibrium of the commensal microbiome [12,13,14] and generates severe hemorrhagic diarrhea in mammals that may cause inappetence, loss of weight and even sudden death [15]. The development and administration of vaccines [16,17,18,19], bactericidal [20,21] and microbiota transplantation [22] remain the main therapeutic strategies against ETEC infection. In the last decade, many cases of antibacterial resistance were reported [23] that have shown the importance of engineering novel antibacterial drugs. In this project, we proposed glycodendrimer-based materials. Glycodendrimers are starburst polymers with predictable molecular weight and organized with a central core surrounded by ramifications ended with reactive groups [24]. Due to the hyperbranched structure and the presence of internal cavities, glycodendrimers are used as carriers for bioactive agents such as drugs or metallic ions. In addition, bioactive molecules (cationic, anionic, polar and non-polar) can be attached at the periphery of the glycodendrimer via the reactive terminal groups. The reactive group could interact with various therapeutic molecules such as DNA, proteins, carbohydrates, or metals according to the biological applications [25]. Further, multivalency is a real advantage for glycodendrimers compared to carbohydrate monomers [26]. Indeed, multivalent carbohydrate–protein target interactions have shown significant advantages, compared to the interactions between carbohydrate monomers and protein targets, due to low binding affinity [27]. For this purpose, several glycodendrimers have been designed to solve the problem of low-affinity carbohydrate–protein interactions [28]. Mannosylated analogs [29] glycodendrimers are considered potential candidates for the treatment of certain strains of *E. coli* by inhibiting bacterial adhesion and the formation of biofilms on the cell surface [30]. Further biological applications of glycodendrimers have been reported [31,32,33].

Due to their anti-fungal and antibacterial properties, glycodendrimers are usually used to inhibit bacterial proliferation [34]; therefore, they constitute a non-neglected route to control bacterial proliferation. Other biopolymers, suitable as carriers for silver and copper are already used in the laboratory to control Gram-negative bacterial proliferation. It was previously shown [35,36] that zero-valent silver and copper hosted by carboxymethyl derivatives are bactericides against non-pathogenic Gram-negative bacteria. We propose now an investigation of the bactericidal activity of glycodendrimers unloaded and loaded zero-valent metals. The mechanism of action of glycodendrimers is not yet completely understood. It is known that certain glycodendrimers carrying metal cations have a hydrophobic tail able to penetrate the cell or facilitate their attachment to the bacterial wall [37,38], changing the membrane permeability by making it porous, and leading to cell lysis. The positive charge of metal cations or of the carbohydrate terminal group and the amphiphilicity alongside the dynamic self-assembling of glycodendrimers are enrolled in their antibacterial activity [39,40,41,42,43,44]. The appearance of pores on the membrane may allow the diffusion of glycodendrimers into the cell, where the carbohydrate terminal groups bind with sulfur and phosphorus-containing proteins, leading to their inactivation and with DNA [45]. Another hypothesis suggests that the antibacterial activity of glycodendrimer loaded with metal nanoparticles results from the release of corresponding ions via the oxidation dissolution process. Metal ions oxidized from corresponding metal-loaded materials mainly interact with thiol groups of various enzymes and proteins, thereby interfering with the respiratory chain and disrupting the bacterial cell wall [46]. In addition, it is known that silver ions are involved in the generation of reactive oxygen species (ROS), which are considered the main cause of most cell deaths via the inactivation of DNA replication and ATP production [47]. In the same way, it was also found that several dendrimers possess antibacterial activity against pathogenic *E. coli* [48].

Metallo-glycodendrimer materials combining the nanoparticle properties due to their nanosize and the alteration of bacteria metabolism due to mannose are expected to be good candidates to overcome microbial proliferation. To the best of our knowledge, there are no reports on the synthesis and on the antibacterial applications of mannosylated dendrimers loaded with zero-valent copper or silver. There are now reports of the synthesis of novel glycodendrimers loaded with zero-valent metals and their applications against enterotoxigenic *E. coli* proliferation (the main cause of swine enteritis). The core of dendrimers was prepared using gallic acid, which has intrinsic antibacterial properties. Mannopyranosides, known to target bacterial pili, were attached as ending reactive groups to form glycodendrimers. In order to enhance the antibacterial activity of these glycodendrimers, zero-valent copper or silver was loaded to form copper-loaded glycodendrimers and silver-loaded glycodendrimers, respectively. The particle size of glycodendrimers-based materials was measured, zero-valent metals entrapped was confirmed, and the antimicrobial activity of metal-free glycodendrimers, copper-loaded glycodendrimers and silver-loaded glycodendrimers against ETEC:F4 was compared with that of 3% hydrogen peroxide.

## 2. Materials and Methods

### 2.1. Materials

All the reagents were used as supplied without any prior purification. The reagents were obtained from Millipore Sigma Canada Ltd. (Oakville, ON, Canada) and Thermo Fisher Scientific (Saint-Laurent, QC, Canada).

Enterotoxigenic *E. coli* Fimbriae 4 (NCBI ID: txid316401), used for bactericidal assays, was from Professor Fairbrother (Pathology and Microbiology Department) at the Veterinary Medicine Faculty of Université de Montréal (St-Hyacinthe, QC, Canada).

### 2.2. Methods

All the organic reactions were carried out using standard methods under an inert atmosphere of nitrogen. The storage of the solvents was carried out using molecular sieves and if necessary, those solvents were bubbled with nitrogen. The monitoring of the reactions was carried out by using thin-layer chromatography (TLC) using silica gel 60 F254 pre-coated plates (E. Merck, Darmstadt, Germany). The TLC was viewed under ultraviolet light at 254 nm or/and chemical stain recipe. The purification was performed by recrystallization or flash (60 Å porosity, 40–63 μm) column chromatography using silica gel (Canadian Life Science, Peterborough, ON, Canada).

The distribution of particle size was measured in water using Dynamic Light Scattering measurements (Malvern, Zetasizer Nano S90, Worcestershire, UK).

^1^H-NMR acquisitions were recorded at 300 MHz, and ^13^C-NMR spectra were recorded at 75 MHz, respectively, on a Bruker spectrometer (300 MHz) (Milton, ON, Canada). All NMR spectra were measured at 25 °C in the described deuterated solvents. The chemical shifts of proton and carbon are reported in parts per million (ppm), and the coupling constants *(J)* are reported in Hertz (Hz). The peaks of the residual protic solvent used for chemical shift calibrations were CDCl_3_ (^1^H, δ 7.27 ppm; ^13^C, δ 77.2 ppm (central resonance of the triplet)), DMSO-d6 (^1^H, δ 2.50 ppm; ^13^C, δ 39.52 ppm) and D_2_O (^1^H, 4.79 ppm and 30.9 ppm for the CH_3_ of the acetone in the ^13^C spectra).

**Synthesis of methyl 3,4,5-tris(hydroxyl)benzoate (2)**. 3,4.5-Trihydroxybenzoic acid (**1**, Gallic acid) (5.0 g, 29.4 mmol) was dissolved in 80 mL of methanol, and 0.5 mL of H_2_SO_4_ was added dropwise. The reaction was refluxed for 7 h and monitored by TLC. When the reaction was completed, the solvent was evaporated to obtain the crude product. The reaction mixture was concentrated and extracted with ethyl acetate (3 × 200 mL). The reaction mixture was then treated with saturated NaHCO_3_ in order to neutralize any acidic traces. The organic layer was dried over sodium sulfate (Na_2_SO_4_) and concentrated in vacuo (rotary evaporator) to provide ester **2** in good yield (5.1 g, 95%); Rf = 0.4 (15% ethyl acetate/hexane). Compound characterization agreed with the literature values [49]. ^1^H-NMR (300 MHz, (CD_3_)_2_SO): δ 9.21 (s, 3H), 6.98 (s, 2H), 3.74 (s, 3H); ^13^C-NMR (75 MHz, (CD_3_)_2_SO): δ 166.8, 146.0, 138.9, 119.9, 109.0 and 52.1 ppm.

**Synthesis of methyl 3,4,5-tris(propargyloxy)benzoate (3).** Compound **2** (1.0 g, 5.4 mmol) was dissolved in 10 mL of dry acetone to which was added potassium carbonate K_2_CO_3_ (5.2 g, 43.4 mmol) followed by the addition of 18-crown-6-ether (57.4 mg, 0.2 mmol) as a co-catalyst. Propargyl bromide (1.6 mL, 43.4 mmol) was next added dropwise, and the reaction was refluxed overnight. The solvent was then evaporated to afford the crude product, which was purified by silica gel column chromatography, which gave compound **3** as a white powder (1.10 g, yield 68%); Rf = 0.4 (15% ethyl acetate/hexane). Compound characterization agreed with literature values [49]; ^1^H-NMR (300 MHz, CDCl_3_): δ 7.41 (s, 2H), 4.77 (s, 6H), 3.86 (s, 3H), 2.54 (s, 2H), 2.46 (s,1H); ^13^C-NMR (75 MHz, CDCl_3_): δ 165.7, 150.8, 140.5, 125.3, 109.3, 78.2, 76.3, 75.9, 75.3, 59.9, 56.6 and 51.9 ppm.

**Synthesis 3,4,5-tris(propargyloxy)benzoic acid (4).** Compound **3** (1.0 g, 3.4 mmol) was dissolved in 40 mL ethanol, followed by the addition of a 10% aqueous solution of KOH (7.5 mL, 752.4 mg, 13.4 mmol) in 7.5 mL water. The reaction was refluxed for 4 h with constant stirring and cooled to room temperature. Subsequently, the reaction mixture was concentrated, and hydrochloric acid was added until pH 1 was obtained. The reaction mixture was extracted with dichloromethane (DCM) and washed with H_2_O. The organic layer was then collected and dried over Na_2_SO_4,_ followed by the solvent evaporation without purification to provide pure compound **4** as a white powder (905 mg, yield 95%); Rf = 0.4 (15% ethyl acetate/hexane). Compound characterization agreed with literature values [49] ^1^H-NMR (300 MHz, (CD_3_)_2_SO): δ 7.40 (s, 2H), 4.91 (d, *J* = 1.2 Hz, 4H), 4.73 (d, *J* = 1.8 Hz, 2H), 3.62 (s, 2H), 3.47 (s,1H); ^13^C-NMR (75 MHz, (CD_3_)_2_SO: δ 167.2, 151.4, 140.1, 126.7, 109.3, 79.6, 79.4, 79.3, 78.8, 59.9 and 56.9 ppm.

**Synthesis of dendrimer core (5).** To a solution of pentaerythritol (25.0 mg, 0.18 mmol) in 10 mL anhydrous DCM (10 mL), compound **4** (271.4 mg, 0.95 mmol), N,N′-dicyclohexylcarbodiimide (DCC) (189.4 mg, 0.92 mmol) and 4-dimethylaminopyridine DMAP (49.4 g, 0.40 mmol) were added. The reaction was refluxed overnight (o.n.). The completion of the reaction was confirmed by TLC, and the reaction mixture was concentrated and purified by silica gel column chromatography to afford compound **5** as a white solid (146.8 mg, yield 67%); Rf = 0.4 (DCM); ^1^H-NMR (100 MHz, CDCl_3_): δ 7.49 (s, 8H), 4.83 (d, *J* = 1.2 Hz, 8H), 4.79 (s, 16H), 4.66 (s, 8H), 2.61 (s, 8H), 2.47 (s,4H); ^13^C-NMR (75 MHz, CDCl_3_): δ 165.2, 151.5, 141.4, 124.7, 109.9, 78.6, 77.9, 76.6, 75.8, 63.3, 60.3, 57.2 and 43.2 ppm.

**Synthesis of triethylene glycol *p*-toluenesulfonate (6).** To a solution of triethylene glycol (16.24 g, 108.15 mmol) in 45 mL of tetrahydrofuran (THF), 6 mL of a 4M aqueous solution of NaOH was added. The reaction mixture was stirred at 0 °C for 1 h, and then a solution of tosyl chloride (2.11 g, 10.82 mmol) in THF (25 mL) was added dropwise using a dropping funnel. Finally, the reaction mixture was stirred at 0 °C for an additional 3 h. The reaction mixture was poured into iced water (200 mL) and extracted with DCM (3 × 200 mL). The organic layer was dried over sodium sulphate and concentrated under vacuo. The crude product was purified by silica gel column chromatography to give compound **6** as a colorless oil (3.29 g, 99%) Rf = 0.4 (30% acetone/DCM). Compound characterization agreed with the literature value [50]. ^1^H-NMR (300 MHz, (CDCl_3_): δ 7.78 (d, *J* = 7.8 Hz, 2H), 7.35 (d, *J* = 8.0 Hz, 2H), 4.15 (t, *J* = 4.8 Hz, 3H), 3.69-3.54 (m, 10H), 2.97 (s, 1H), 2.44 (s, 1H); ^13^C-NMR (75 MHz, (CDCl_3_): δ 145.0, 132.7, 127.9, 72.5, 70.6, 70.1, 69.3, 68.6, 61.5 and 21.6 ppm.

**Synthesis of 2-[2-(2-(2-Tosyloxy-ethoxy)-ethoxy)-ethyl]2,3,4,6-tetra-O-acetyl-α-D-mannopyranoside (8).** This compound was prepared according to a slight modification of the literature procedure [51]. To a solution of known mannose pentaacetate (**7**) (1.00 g, 2.56 mmol) [51] in anhydrous DCM (10 mL) was added triethylene glycol *p*-toluene sulfonate (1.8 g, 5.98 mmol) (**6**) and the mixture was stirred at room temperature for 1h. The reaction mixture was then cooled to 0 °C in an ice bath. Boron trifluoride etherate (BF_3_OEt_2_, 3.8 g, 10.79 mmol) was next added dropwise under a nitrogen atmosphere, and the reaction was stirred for 2 h. The reaction mixture was next heated at 40 °C overnight until complete glycosylation. The crude product was extracted with DCM and treated with NaHCO_3_. The organic layer was dried over Na_2_SO_4,_ followed by solvent evaporation. The product was purified by silica gel column chromatography to afford compound **8** as a colorless oil (1.0 g, yield 60%). Rf = 0.35 (EtOAc/Hexane, 1:4).^1^H-NMR (300 MHz, CDCl_3_) δ7.80 δ (7.75 (d, *J* = 8.3 Hz, 2H), 7.31 (d, *J* = 8.0 Hz, 2H), 5.32-5.21 (m, 3H), 4.82 (d, *J* = 1.6 Hz, 1H), 4.25 (dd, *J* = 12.3, 5.1 Hz 1H), 4.14-4.08 (m, 2H), 4.07-4.02 (m, 1H), 3.76-3.54 (m, 11H), 2.12 (s, 3H), 2.11 (s, 3H), 2.05 (s, 3H), 1.99 (s, 3H), 1.94 (s, 3H).^13^C-NMR (75 MHz, CDCl_3_) δ 170.2, 169.6, 169.4, 169.3, 144.4, 132.6, 129.4, 127.5, 97.3, 70.3, 70.2, 69.6, 69.1, 68.9, 68.6, 68.3, 68.0, 66.9, 65.7, 62.0, 21.2, 20.4, 20.3, 20.3, 20.2.

**Synthesis of 2-[2-(2-(2-Azido-ethoxy)-ethoxy)-ethyl]2,3,4,6-tetra-O-acetyl-α-D-mannopyranoside (9)** A solution of the compound **8** (854 mg, 1.7 mmol) in DMF (15 mL) was stirred under a nitrogen atmosphere then sodium azide (884 mg, 13.6 mmol) was added and the reaction mixture was stirred at 80 °C for overnight. The reaction mixture was diluted in EtOAc and washed with saturated solution of sodium chloride (brine). The organic layer was dried over Na_2_SO_4,_ followed by the solvent evaporation to afford compound **9** as a colorless oil (800 mg, yield 93%), Rf = 0.35 (EtOAc/Hexane, 1:4). Compound characterization agreed with the literature values [52]. ^1^H-NMR (300 MHz, CDCl_3_) δ 5.39-5.26 (m, 3H), 4.88 (d, *J* = 1.6 Hz, 1H), 4.29 (dd, *J* = 12.4, 5.2 Hz, 1H), 3.71-3.67 (m, 2H), 3.91-3.75 (m, 1H), 3.76-3.60 (m, 9H), 3.42-3.38 (t, *J* = 4,8 Hz, 2H), 2.16 (s, 3H), 2.11 (s, 3H), 2.05 (s, 3H), 2.00 (s, 3H). ^13^C-NMR (75 MHz, CDCl_3_) δ 170.4, 169.8, 169.7, 169.5, 97.5, 77.2, 69.8, 69.7, 69.3, 68.8, 67.1, 50.4, 20.6, 20.5, 20.4, 20.4.

**Synthesis of peracetylated glycodendrimer (10).** To a solution of compound **5** (25.0 mg, 0.02 mmol) and excess compound **9** (194.4 mg, 0.37 mmol, 18.5 equiv., 1.5 equiv/alkyne groups) in H_2_O/THF (4 mL, *v*/*v*) was added sodium ascorbate (29.3 mg, 0.24 mmol) and Cu(OAc)_2_ (41.6 mg, 0.17 mmol). The solution was heated at 50 °C for 12 h and then cooled to room temperature until complete conversion of the alkyne used as limiting reagent (overnight). The reaction mixture was extracted with EtOAc, which was then treated with 5% ethylene diamine tetraacetic acid (EDTA), water, brine and dried over Na_2_SO_4_. The organic phase was concentrated, and the residue was purified by silica gel column chromatography to afford the protected glycodendrimer. Yield (93.8 mg, 64%); Rf = 0.4 (DCM); ^1^H-NMR (300 MHz, CDCl_3_): δ 8.01 (s, 8H), 7.99 (s, 4H), 7.49 (d, *J* = 1.2 Hz, 12H), 5.33-5.20 (m, 60H), 4.85 (s, 12H), 4.74 (s, 8H), 4.57 (s, 36H), 4.29-4.23 (m, 12H), 4.14-4.05(m, 12H), 3.90-3.80 (m, 12H), 5.33-5.20 (m, 96H), 2.61 (s, 8H), 2.47 (s, 4H); ^13^C-NMR (75 MHz, CDCl_3_): δ 170.3, 169.7, 169.6, 169.4, 164.9, 151.7, 124.8, 124.3, 108.9, 97.3, 70.3, 70.1, 69.6, 69.2, 68.7, 68.1, 65.7, 62.0, 49.9, 20.6, 20.4, 20.4, 20.4.

**Synthesis of unprotected glycodendrimer (11)** Peracetylated manno-dendrimer **10** (70.0 mg, 0.01 mmol) was trans-esterified under classical Zemplén conditions in dry MeOH (3 mL) containing a solution of 1M sodium methoxide (MeOH, pH 8.5). The reaction mixture was stirred at room temperature until the starting material completely disappeared, as confirmed by a single spot on TLC at the baseline. The reaction mixture was neutralized by the addition of a cationic ion-exchange resin (H^+^), filtered, evaporated under vacuo, and the residue was lyophilized to obtain the final unprotected glycodendrimer **11** (45.6 mg) in 89%yield. ^1^H-NMR (300 MHz, D_2_O): δ 7.49 (s, 8H), 4.91 (d, *J* = 1.2 Hz, 4H), 4.79 (s, 16H), 3.89 (dd, *J* = 10.5, 5.7 Hz, 48H), 3.78 (dd, *J* = 12.5, 6.9 Hz, 1H), 3.69 (dd, *J* = 12.4, 3.5 Hz, 1H), 3.58 (dd, *J* = 16.9, 7.6 Hz, 1H), 3.51-3.45 (m, 84H); ^13^C-NMR (75 MHz, D_2_O): δ 165.1, 151.5, 141.4, 124.7, 109.9, 78.6, 77.9, 76.6, 75.8, 59.9, 63.2, 60.3, 57.2 and 43.2 ppm.

**Metal-entrapment in glycodendrimer.** The cation-loaded dendrimers were obtained by adding 15 mg of the glycodendrimer to 10 mL aqueous solutions of either 0.0005 mol/L Cu(OAc)_2_ or 0.0005 mol/L AgNO_3_ under vigorous stirring at room temperature for 2 h according to Noori et al. with minor modifications [35]. Then, to the obtained cation-containing glycodendrimers (**11-Cu^2+^** or **11-Ag^+^**) were added 10 mL of 0.001 mol/L NaBH_4_ solution and the mixtures were stirred for 6 h at room temperature to afford zero-valent CuNP-loaded glycodendrimer (**11-Cu^0^**) and AgNP-loaded glycodendrimer (**11**-**Ag^0^**). Metals zero-valent were obtained by reduction with NaBH_4_ in similar conditions as described by Noori et al. [35] with the identification of zero-valent metals by XPS spectroscopy.

**Effect of glycodendrimers on ETEC proliferation.** Enterotoxigenic *E. coli* fimbriae 4 (ETEC:F4) 1 × 10^7^ CFU/mL were treated with various concentrations of metal-glycodendrimers (0–0.075 mg/mL of broth) loaded or not with metal nanoparticles. For the negative control, the ETEC:F4 bacteria were treated with Luria–Bertani (LB) broth from Becton, Dickinson and Company (Sparks, MD, USA). The samples at a final volume of 10 mL were incubated for 24 h at 37 °C, 100 RPM and the optical density at 600 nm (OD_600nm_) was measured in a polystyrene cuvette of 10 mm path length by Biochrom Libra S50 UV-Vis spectrophotometer (Biochrom US, Holliston, MA, USA). The bactericidal activity of metal-glycodendrimers was also evaluated by inhibition zone diameter of 1 mg powder of glycodendrimers on LB agar Petri dishes, previously inoculated with ETEC:F4. The disc images were acquired after 24 h incubation at 37 °C, analyzed by ImageJ2 software (version 2, Madison, WI, USA) and the diameters of the inhibition zones were reported in centimeters.

**Quantification of protein released by ETEC:F4 treated with metal-loaded glycodendrimers for 24 h at 100 RPM.** In order to understand the metallo-glycodendrimers mechanisms on ETEC:F4 death, proteins, DNA, RNA and oligonucleotides released from bacteria were measured in the LB broth. A volume of 5 mL was taken from the mixture of bacteria previously incubated with corresponding glycodendrimers for 24 h. The sample was centrifugated at 800 RPM × 10 min at 4 °C, and the supernatant was collected to quantify bacteria-released contents. The Bradford method [53] was used to assay proteins contained in the supernatant by absorbance measurement at 595 nm.

**DNA, RNA and oligonucleotide assay.** A volume of 0.5 mL of the supernatant collected from bacteria culture centrifugation (800 RPM × 10 min at 4 °C) was transferred to spin columns (Bio-Rad Laboratories Ltd., Mississauga, ON, Canada) inserted into a 2 mL Eppendorf used as a collection tube and centrifuged at 6000×*g* for 2 min at 4 °C according to the protocols of nucleic acids separation of the manufacturer. The centrifugation rounds were repeated three times. For each round, new spin columns and new Eppendorf vials are used to collect the elution solutions. The DNA, RNA and oligonucleotide concentrations were acquired by NanoDrop™ 2000/2000c Spectrophotometer (Thermo Fisher Scientific, Mississauga, ON, Canada) based on the 260/280 or 260/230 ratios following the protocols of the manufacturer.

**Biofilm assay.** ETEC was identified as a biofilm-forming species. Biofilm is a critical factor for microbial survival and antibiotic resistance. ETEC:F4 biofilm formation was followed in polystyrene 96-well plates. Bacteria, at a concentration of 10^7^ CFU/mL treated or not with glycodendrimer-based materials 0.005 mg/mL (a concentration close to the IC_50_ of less toxic glycodendrimeric materials), were grown at 37 °C for 24 h in Luria–Bertani broth. After incubation, bacteria were gently washed three times with 200 μL of PBS in order to remove non-adhered bacteria. The attached bacteria, representing the matrix components of biofilm, were stained with 100 μL of a 0.1% solution of crystal violet for 30 min at room temperature, according to Mintzer et al.’s protocol [54]. Crystal violet salt was then solubilized by the addition of 30% glacial acetic acid and incubated at room temperature for 10–15 min. The absorbance was measured by a Biochrom EZ Read 800 microplate reader (Biochrom US, Holliston, MA, USA) at 570 nm, and data were reported as mean ± standard deviation. All experiments were repeated three times.

## 3. Results and Discussion

The glycodendrimer was conceived as based on antibacterial constituent elements exhibiting inner antibacterial activity. Thus, gallic acid, which is a natural polyphenol that is found in plants, was chosen for the synthesis route of the glycodendrimer core. Gallic acid is known to have strong antibacterial properties on its own and can affect irreversibly the *E. coli membrane* [55]. Additionally, mannose was also chosen because it has been well established that *E. coli* possesses a carbohydrate-binding protein at the tip of their pili associated with FimH, which recognizes α-D-mannopyranoside glycoconjugates on the host cell membranes [29]. Hence, mannoside NPs can be envisaged as targeting devices. Therefore, combining the above two components into a single entity presented into a multivalent architecture (mannosylated glycodendrimer) was hypothesized to greatly enhance fighting *E. coli* bacterial infections.

### 3.1. Synthesis of the Core Structure

**Scheme 1:**
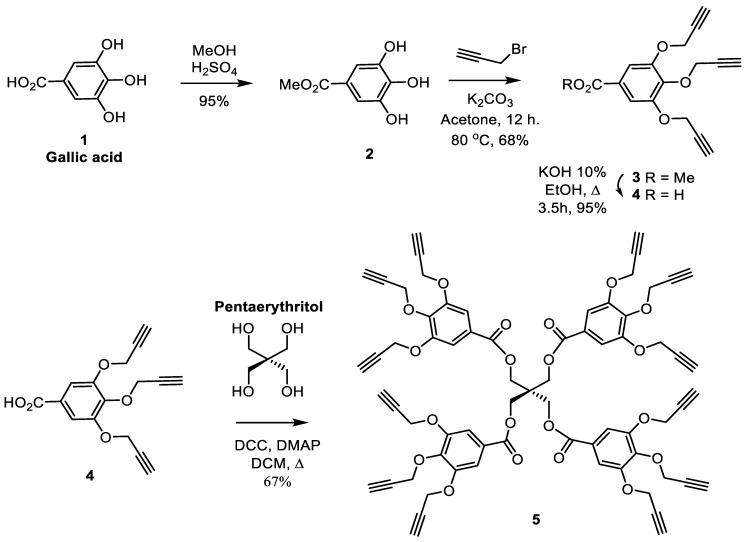
Synthesis of the key dendritic pentaerythritol scaffold harboring four gallic acid residues covered with 12 propargyl functions used for the preparation of multivalent mannosylated dendrimer by a copper-catalyzed azide–alkyne cycloaddition (CuAAC).

The propargylated core structure was built from pentaerythritol and a propargylated gallic acid derivative (Scheme 1). First, methyl gallate (**2**) [49] was obtained from the 3,4,5-trihydroxybenzoic by using Fischer esterification in order to protect the acid functionality. Then, the methyl ester was functionalized with alkyne groups in the periphery, and the propargylation reaction, including phenol groups, led to compound **3.** Then, the obtained compound **3** was treated with aqueous KOH to hydrolyze the ester and give the compound **4** (3,4,5-tris(propargyloxy)benzoic acid) (Scheme 1) [56].

A dendrimer scaffold harboring 12 propargyl groups was chosen in order to allow a first generation with 12 mannoses on the glycodendrimer periphery, so 48 hydroxyl groups only for generation 1, which is remarkable given that the comparable number of hydroxyl groups is generally obtained from higher generations for commercial poly(amidoamine) dendrimers [57]. The propargylated gallic acid (**4**) was reacted according to the Steglich reaction with pentaerythritol using DCC as a coupling agent and Dimethyl aminopyridine (DMAP) as a nucleophile to afford the tetravalent glycodendrimer core (**5**) by esterification coupling. According to the literature, the Steglich reaction [58] carried out at room temperature is known to take several days. The ^1^H-NMR confirmed the presence of the propargylic protons at around 2.5 ppm (Figure S1), and the product was confirmed by ^13^C-NMR (Figure S2).

### 3.2. Synthesis of the Carbohydrate for Core Branching

**Scheme 2:**
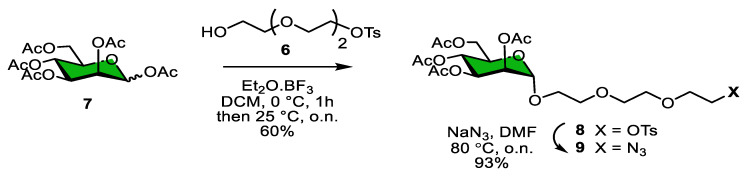
Synthesis of the peracetylated mannosylated azide precursor **9**.

For the sugar moiety, our approach aimed to synthesize an ethylene glycol linker using triethylene glycol: a Food and Drug Administration (FDA)-approved agent. A monotosylation of triethylene glycol was carried out under cold conditions to obtain the desired product without any by-product. The D-mannose was also treated with acetic anhydride in pyridine for the acetylation of the mannose hydroxyl groups to afford the 1,2,3,4,6-penta-O-acetyl-α/β-D-mannopyranose (**7**) which was glycosylated with triethylethylene glycol p-toluenesulfonate (**6**) using Lewis acid (BF_3_-Et_2_O) to give the 2-(2-(2-(2-Tosyloxy-ethoxy)-ethoxy)-ethyl)2,3,4,6-tetra-O-acetyl-α-D-mannopyranoside (**8**). Finally, by carrying out a nucleophilic substitution, the tosylate group of compound **8** was converted into an azide group present at the focal point of our linker. (Scheme 2). ^1^H-NMR showed the disappearance of the aromatic protons of the tosylate group at 7.77 and 7.32 ppm, as expected.

### 3.3. Synthesis of Mannosylated Glycodendrimer

The Copper-assisted Azide–Alkyne Cycloaddition (CuAAC), according to Sharpless et al. [55,59] is known to be efficient, simple, selective and frequently used in dendrimer synthesis.

**Scheme 3:**
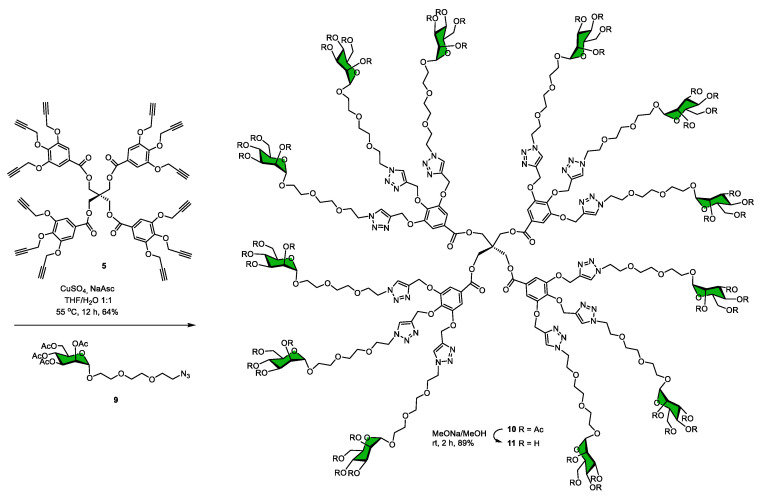
Synthesis of mannosylated glycodendrimer **11** which is endowed with 12 α-D-mannopyranoside residues at the periphery. The synthesis was accomplished according to the well-established “click chemistry” (CuAAC) between the propargylated core **5** and the mannosylated sugar azide **9**, followed by a selective Zemplén reaction.

The ^1^H-NMR of **10** gives some indications of the reaction accomplishment. First, the disappearance of the two asymmetrical propargyl proton peaks at δ 2.61 ppm and δ 2.47 ppm, as well as the appearance of the triazole peaks at δ 8.01 and δ 7.99 ppm, unambiguously confirmed the completion of the click reaction (Figure S3). The ^1^H-NMR analysis was also supported by ^13^C-NMR (Figure S4). The deprotection of the peracetylated glycodendrimer **10** (Scheme 3) was carried out under mild Zemplén trans-esterification conditions (NaOMe, MeOH, pH 8.5), which allowed maintaining the inner (protected) gallate esters. The whole disappearance of the acetate peaks (144 protons) in the ^1^H-NMR spectra of the resulting mannodendrimer **11** (Scheme 3) appeared between δ 1.9 and 2.2 ppm (Figure S5). The unprotected glycodendrimer (**11**) identified by ^13^C-NMR (Figure S6) spectra confirmed the completion of the reaction.

### 3.4. Characterization of Nanoparticles of Cu- and Ag-Loaded Glycodendrimers

The relevant antibacterial properties of zero-valent copper and silver nanoparticles hosted by biopolymers are already documented [35] and these zero-valent metals were used in this project to increase the antibacterial activity of the glycodendrimers. The metal ions from Cu(OAc)_2_ and AgNO_3_ were first uniformly distributed in the dendrimeric dispersion and then reduced with NaBH_4_ to generate the corresponding zero-valent metals, homogenously entrapped by glycodendrimers. A DLS study of metal-glycodendrimeric materials showed particle sizes smaller than 100 nm for the metal-loaded glycodendrimers. The medium size for the D:Cu was slightly lower (41–64 nm) than for the D:Ag (61–80 nm), whereas the medium size of unloaded glycodendrimers (D) was 64–65 nm, suggesting no major impact of metal loading on the size of dendrimers. In addition, polydispersity indexes of 0.42 ± 0.03, 0.47 ± 0.03 and 0.33 ± 0.01 were obtained for D, D:Cu and D:Ag, respectively.

### 3.5. Bactericidal Activity of Metal-Glycodendrimers

**Figure 1:**
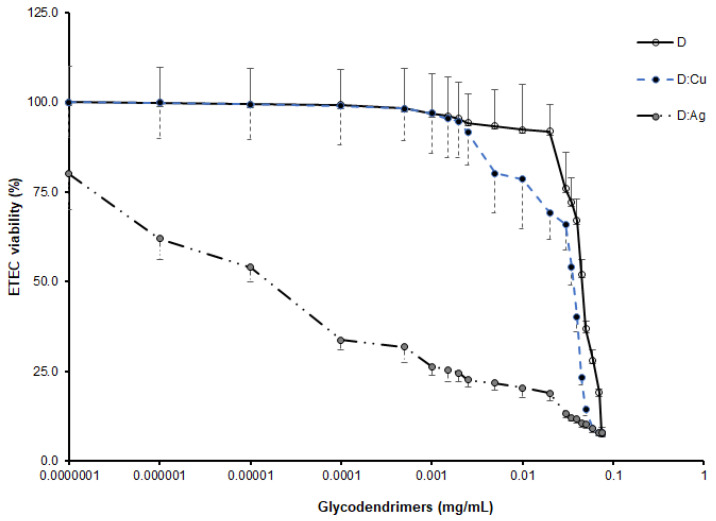
Effect of metal-glycodendrimers on ETEC:F4 bacteria. Evaluation by OD at 600 nm of survival of enterotoxigenic *E. coli* fimbriae 4 (ETEC:F4) from LB broth containing glycodendrimer-based compounds at different concentrations. Data are triplicate of three different experiments and are represented by mean ± SD.

**Figure 2:**
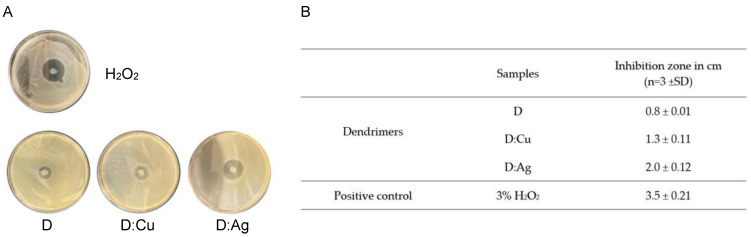
(**A**) Diffusimetric profile of the Anti-ETEC:F4 materials. Effect of glycodendrimer (D), of glycodendrimer complexed with copper (D:Cu) and of glycodendrimer complexed with silver (D:Ag) was evaluated on agar-LB gel. A solution of 3% H_2_O_2_ was used as a positive control. (**B**) Average diffusion diameters as growth inhibition of bacteria induced by 1 mg of glycodendrimer–based materials and by 10 μL of 3% H_2_O_2_. The experiments were repeated three times, and data represented mean ± SD.

The enterotoxigenic *Escherichia coli* fimbriae 4 (ETEC:F4) was selected to evaluate the bactericidal effect of zero-valent metal entrapment in glycodendrimers. The decrease in ETEC survival measured by optical density at 600 nm was inversely proportional to the increase in glycodendrimer concentrations (Figure 1). The minimal inhibitory concentrations (MIC) were 2.0, 1.5 and 1.0 × 10^−4^ µg/mL for glycodendrimers (D), glycodendrimers loaded with copper (D:Cu) and glycodendrimers loaded with silver (D:Ag), respectively. These values were markedly lower (10–1000 folds) than MIC of anti-ETEC:F4 agents used in veterinary and human medicine. In the same way, the half inhibitory concentrations of bacteria growth (IC_50_) were 4.5 × 10^1^, 3.5 × 10^1^ and 1.0 × 10^−2^ µg/mL for D, D:Cu and D:Ag, respectively (Figure 2, inserted table). These data support our hypothesis that the addition of mannose (antimicrobial carbohydrate) as terminal groups and the loading of glycodendrimers by zero-valent copper and silver nanoparticles, inhibited the ETEC:F4 proliferation and enhanced the bactericidal activity of designed dendrimer, with the highest activity for silver zero loaded glycodendrimers (Table S1).

The inhibition of ETEC:F4 bacteria proliferation at metal glycodendrimer-dependent concentrations (Figure 1) correlated with the inhibition diameters (Figure 2A,B) confirmed the bactericidal activity of glycodendrimeric materials loaded or not with zero-valent metal (Table S1). Additional experiments were conducted with Phosphomycin, Gentamycin and Kanamycin and compared with our unloaded (D) and metal-loaded mannodendrimers (D:Cu°, D:Ag°). The choice of Phosphomycin, Gentamycin and Kanamycin was based on the fact that these antibiotics are currently used to treat enterotoxigenic *E. coli* (ETEC). The results expressed as diffusion diameters showed our materials D:Ag with a moderately higher bactericidal efficacy than those of Phosphomycin and Gentamycin and comparable with that of Kanamycin. Differently, the D and D:Cu materials presented a lower bactericidal efficacy (Table S1).

At concentrations of 10 mM and higher, H_2_O_2_ may react with DNA and other macromolecules of bacteria and generate the highly reactive and damaging hydroxyl radical (HO•) via the Fenton reaction [60]. The choice of 3% H_2_O_2_ (~1M) as positive control is based on the fact that, at this much higher concentration (around 100 times greater than that mentioned as cytotoxic), it is currently used as a disinfectant in slaughterhouses and for farm equipment due to its potential to kill all kinds of cells [61,62] including *E. coli* [60,63,64].

A common mechanism of antibacterial agents is the disruption of bacterial walls or the modification of nucleotidic contents. In order to confirm the metal glycodendrimer action on the loss of integrity of bacteria wall, the proteins released in the LB broth from ETEC:F4 treated or not with various concentrations of metal-glycodendrimer (0–0.03 mg/mL) for 24 h was assayed by Bradford method [53] based on the absorbance measurement at 595 nm, whereas DNA, RNA and oligonucleotide concentrations were acquired according to the protocol of the nanodrop spectrophotometer manufacturer.

**Figure 3:**
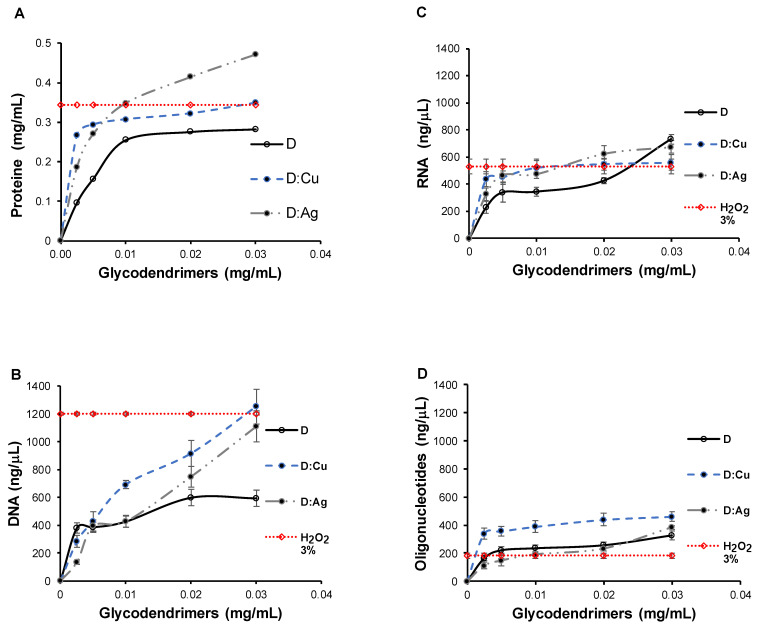
Quantification of released bioactive ETEC:F4 contents. Bacteria were treated with D, D:Cu, D:Ag or with 3% H_2_O_2_ (added in the absence of glycodendrimeric materials). The quantification of protein (**A**) was carried out by the Bradford method, whereas DNA (**B**), RNA (**C**) and oligonucleotides (**D**) concentrations were acquired by the protocol of the manufacturer of NanoDrop spectrophotometer (n = 3, mean ± SD).

To quantify released proteins (Figure 3A), DNA (Figure 3B), RNA (Figure 3C) and oligonucleotides (Figure 3D), the ETEC:F4 (1 × 10^7^ CFU/mL) was treated with various concentrations (0–0.03 mg/mL) of glycodendrimer-based materials. It was found that the bacterial cytoplasmic content increases in LB broth with the concentration of glycodendrimeric materials (Figure 3), confirming a loss of integrity of ETEC:F4 membrane during the treatment and the bactericidal activity of glycodendrimers (D), copper-loaded glycodendrimers (D:Cu) and silver-loaded glycodendrimers (D:Ag).

Supposing a loss of ETEC membrane integrity, the treatment with D, D:Cu and D:Ag generated an increase in protein concentration in LB broth, but this was not proportional to the increasing concentrations of glycodendrimeric materials. The plot obtained from the releasing of proteins versus the concentration of the glycodendrimers (0–0.03 mg/mL) represented two phases: the first one faster, with a high slope, correlated with a higher amount of ETEC:F4 in the LB broth and with an unstable rate of protein release from bacteria, and the second slower phase, with a low or moderately low slope, which might be related to protein release at a stable rate.

Coomassie brilliant blue R250 contains two negatively charged sulphated groups, able to establish electrostatic interactions with cationic amino acids of proteins. No metal-dependent interference was found between Coomassie R250 dye with Cu(OAc)_2_ and AgNO_3_ salts, or with copper- and silver-loaded glycodendrimers. So, proteins were accurately detected in the broth and were from bacteria.

The values of bioactive agents obtained with the 3% H_2_O_2_ positive control were constant for each sample due to the identical concentration. Supposing that each of the three glycodendrimers might disrupt the ETEC wall and liberate its content into the LB broth, the bactericidal effects of each glycodendrimer were also evaluated by assays of released DNA, RNA and oligonucleotides. Among the investigated agents, D:Ag appeared, as expected, to be the most bactericidal material. In opposite to our attempts, the DNA and oligonucleotides released from bacteria cytosol by D:Ag treatment was the lowest, suggesting interactions between D:Ag and DNA and between D:Ag and oligonucleotides as observed with many antibacterial agents able to interact and modify nucleic acids and oligonucleotides of bacteria [65].

**Figure 4:**
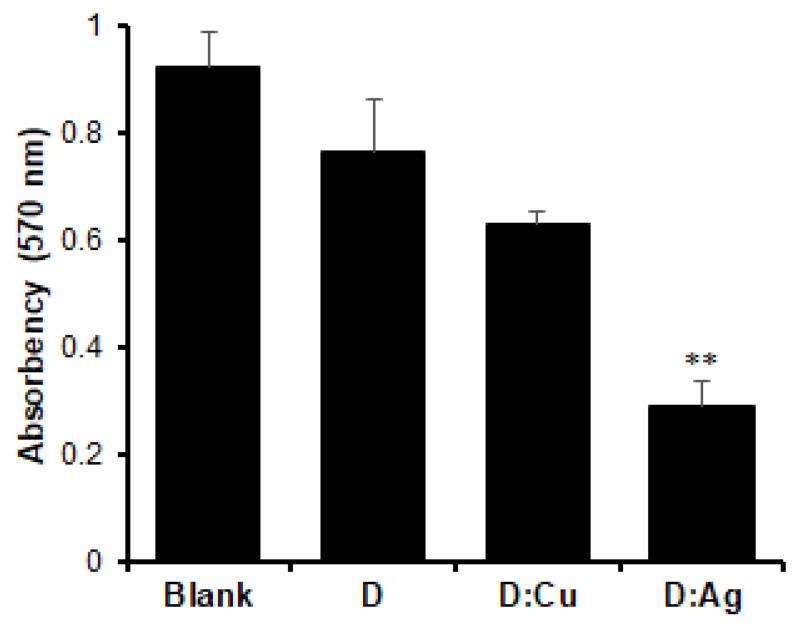
Metal-glycodendrimers inhibit the formation of biofilm by enterotoxigenic *Escherichia coli* fimbriae 4 (ETEC:F4) was treated with 0.005 mg/mL of glycodendrimer (D), glycodendrimer loaded with copper (D:Cu) and glycodendrimer loaded with silver (D:Ag). The biofilm formation assayed by crystal violet colorimetry decreased at treatment with metal glycodendrimers. The untreated bacteria were representative of the blank. (n = 3, mean ± SD). ** *p* < 0.001.

The capacity to form biofilm was investigated and compared between the different glycodendrimeric materials using the crystal violet method indirectly related to the presence of colored solution with absorbency at 570 nm. Treatment of bacteria with 0.005 mg/mL D, D:Cu and D:Ag inhibited the capacity to form the biofilm in comparison to the untreated ETEC:F4 (Figure 4 and Figure S7). The absorbency resulting from the reaction of crystal violet and the biofilm generated by bacteria grown with D, D:Cu and D:Ag was substantially reduced by approximately 20, 30 and 70%, respectively, when compared to the untreated (blank), considered as 100%.

These results corroborated those obtained for bactericidal activity assay (Figure 1), suggesting that D, D:Cu and D:Ag might inhibit the bacteria-to-bacteria interaction, interfere with bacteria information transmission, promote bacteria aggregation or prevent adhesion of bacteria to the surface of the well plate [66,67,68].

## 4. Conclusions

This report showed the bactericidal activity of metal glycodendrimers formed with gallic acid in the core and containing mannose as the carbohydrate-reactive terminal group. This glycodendrimeric material can host zero-valent metals (Cu^0^ and Ag^0^) and appears as a potent antibacterial candidate. More precisely, this type of glycodendrimer showed activity against enterotoxigenic *E. coli* fimbriae 4 (ETEC:F4) bacteria with MIC lower than those of several common antibiotics. The entrapment of zero-valent metal increased the bactericidal effect of glycodendrimeric materials, and the Ag-loaded glycodendrimer (D:Ag) was the most potent bactericidal agent compared to Cu-loaded glycodendrimer (D:Cu) and metal-free glycodendrimer (D). These results show the synthesized glycodendrimeric materials as new bactericidal agents against Gram-negative bacteria such as enterotoxigenic *E. coli* Fimbriae 4. The bactericidal activity could be explained by bacteria wall disruption and by the release of bacteria content in the culture broth. In addition, D, D:Cu and D:Ag were able to inhibit the ETEC:F4 capacity to form biofilm. The bactericidal effects of D:Ag were higher than those of phosphomycin and gentamycin, currently used antibiotics.

## Data Availability

Data are contained within this article and supplementary materials.

## Supplementary Materials

The following supporting information can be downloaded at https://www.mdpi.com/article/10.3390/microorganisms12050966/s1. Figure S1. ^1^H-NMR at 300 MHz, in CDCl_3_ of **5**; Figure S2. ^13^C-NMR at 75 MHz, in CDCl_3_ of **5**; Figure S3. ^1^H-NMR (300 MHz, CDCl_3_) of **10**; Figure S4. ^13^C-NMR (75 MHz, CDCl_3_) of **10**; Figure S5. ^1^H-NMR (300 MHz, D_2_O) of **11**; Figure S6. ^13^C-NMR (75 MHz, D_2_O) of **11;** Figure S7: Biofilm production capabilities of *Escherichia coli* enterotoxigenic fimbriae 4 (ETEC:F4) by crystal violet Assay; Table S1: Antibiotics effect on ETEC:F4.

## Abbreviations

ATP	Adenosine triphosphate
BSA	Bovine Serum Albumin
(CD_3_)_2_SO	Deuterated dimethyl sulfoxide
CDCl_3_	Deuterated chloroform
^13^C-NMR	Carbon-13 Nuclear Magnetic Resonance
COCH_3_	Acetyl group
CRh6G	Calcozine Red 6G
CuAAC	Copper(I) azide–alkyne cycloaddition reaction
Cu(OAc)_2_	Copper(II) acetate
D	Glycodendrimers
d	Doublet
dd	Doublet of doublet
D:Ag	Glycodendrimers loaded with silver
D:Cu	Glycodendrimers loaded with copper
DCC	N, N′-dicyclohexylcarbodiimide
DCM	Dichloromethane
DLS	Dynamic Light Scattering
DMAP	(Dimethylamino)pyridine
DMF	N, N-dimethylformamide
DMSO-d6	Deuterated dimethyl sulfoxide
2D NMR	Two Dimension Nuclear Magnetic Resonance
EtOAc	Ethyl acetate
EDA	Ethylene diamine
EDTA	Ethylenediaminetetraacetic acid
ETEC:F4	Enterotoxigenic *Escherichia coli* fimbriae 4
IC_50_	Half Inhibitory Concentration
J	Coupling constants
K_2_CO_3_,	Potassium carbonate
MA	Methyl acrylate
MeOD	Deuterated methanol
MIC	Minimum Inhibitory Concentration
Na_2_SO_4_	Sodium sulfate
NPs	Nanoparticles
ppm	Parts per million
ROS	Reactive Oxygen Species
THF	Tetrahydrofuran
TLC	Thin-Layer Chromatography
