# Supplementary material for: Metallo-Glycodendrimeric Materials against Enterotoxigenic Escherichia coli
Source: Microorganisms. 2024 May 11;12(5):966. doi: 10.3390/microorganisms12050966 (PMC11124148; doi:10.3390/microorganisms12050966)
Supplement: Supplementary file 1 [file microorganisms-12-00966-s001.zip › microorganisms-2930880-supplementary.pdf]

Supplementary materials

Metallo-Glycodendrimeric Materials against Enterotoxigenic *Escherichia coli*

Aly El Riz [1,†], Armelle Tchoumi Neree [2,3,†], Leila Mousavifar [1], René Roy [1], Younes Chorfi [2,3] and Mircea Alexandru Mateescu [1,3,*]

[1] Department of Chemistry, Université du Québec à Montréal, Succ. Centre-Ville, P.O. Box 8888,
Montréal, QC H3C 3P8, Canada; el_riz.aly@courrier.uqam.ca (A.E.R.);
leilyanmousavifar@gmail.com (L.M.);
roy.rene@uqam.ca (R.R.)

[2] Department of Veterinary Biomedicine Sciences, Faculty of Veterinary Medicine, Université de Montréal,
St-Hyacinthe, QC J2S 2M2, Canada; armelle.tchoumi.neree@umontreal.ca (A.T.N.);
younes.chorfi@umontreal.ca (Y.C.)

[3] Centre de recherche en infectiologie porcine et avicole (CRIPA), Université de Montréal,
St-Hyacinthe, QC J2S 2M2, Canada

* Correspondence: mateescu.m-alexandru@uqam.ca; Tel.: +1-(514)-987-4319

† These authors contributed equally to this work as first author.

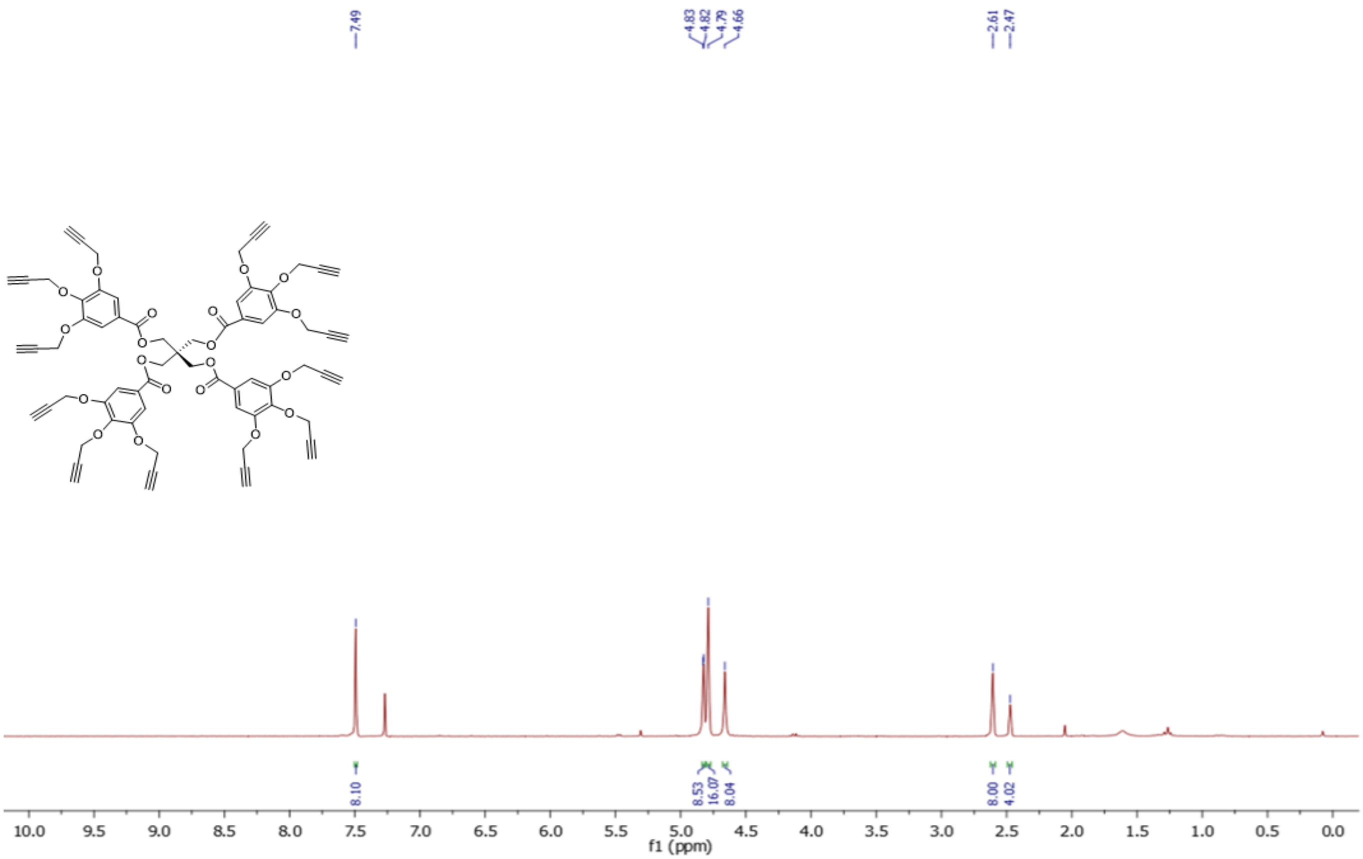

Figure S1. ^{1}H-NMR (300 MHz, $CDCl_3$) of compound 5

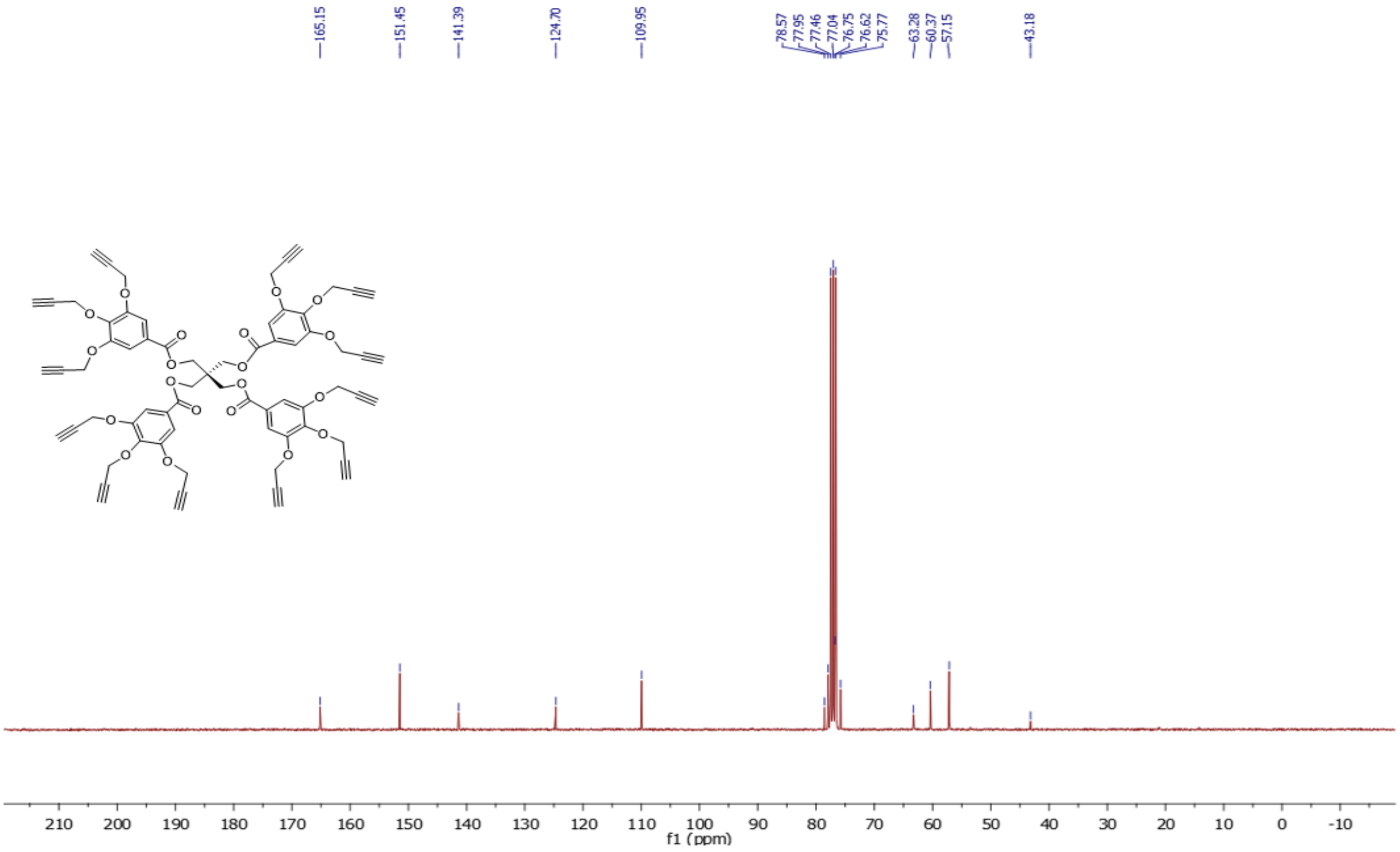

Figure S2. ^{13}C-NMR (75 MHz, $CDCl_3$) of compound 5

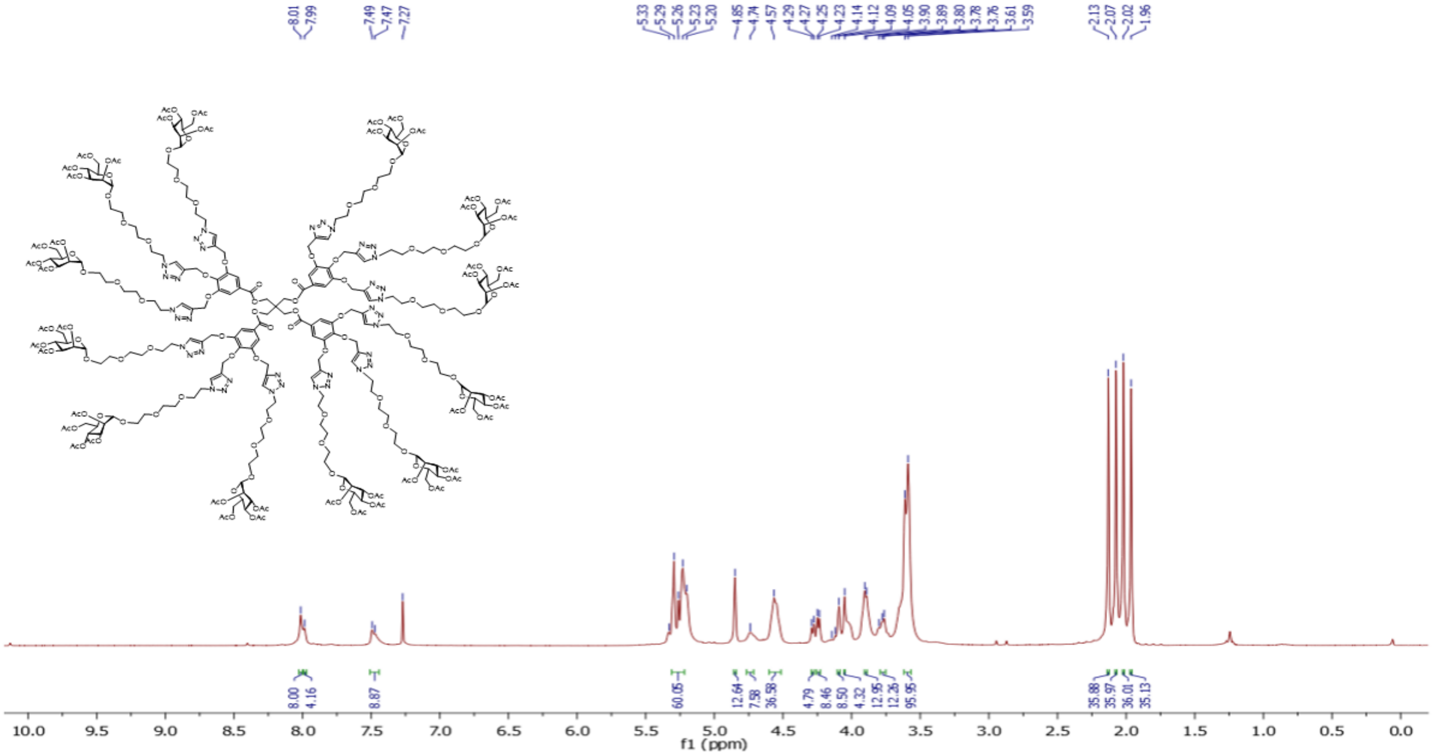

Figure S3. ^{1}H-NMR (300 MHz, $CDCl_3$) of compound 10

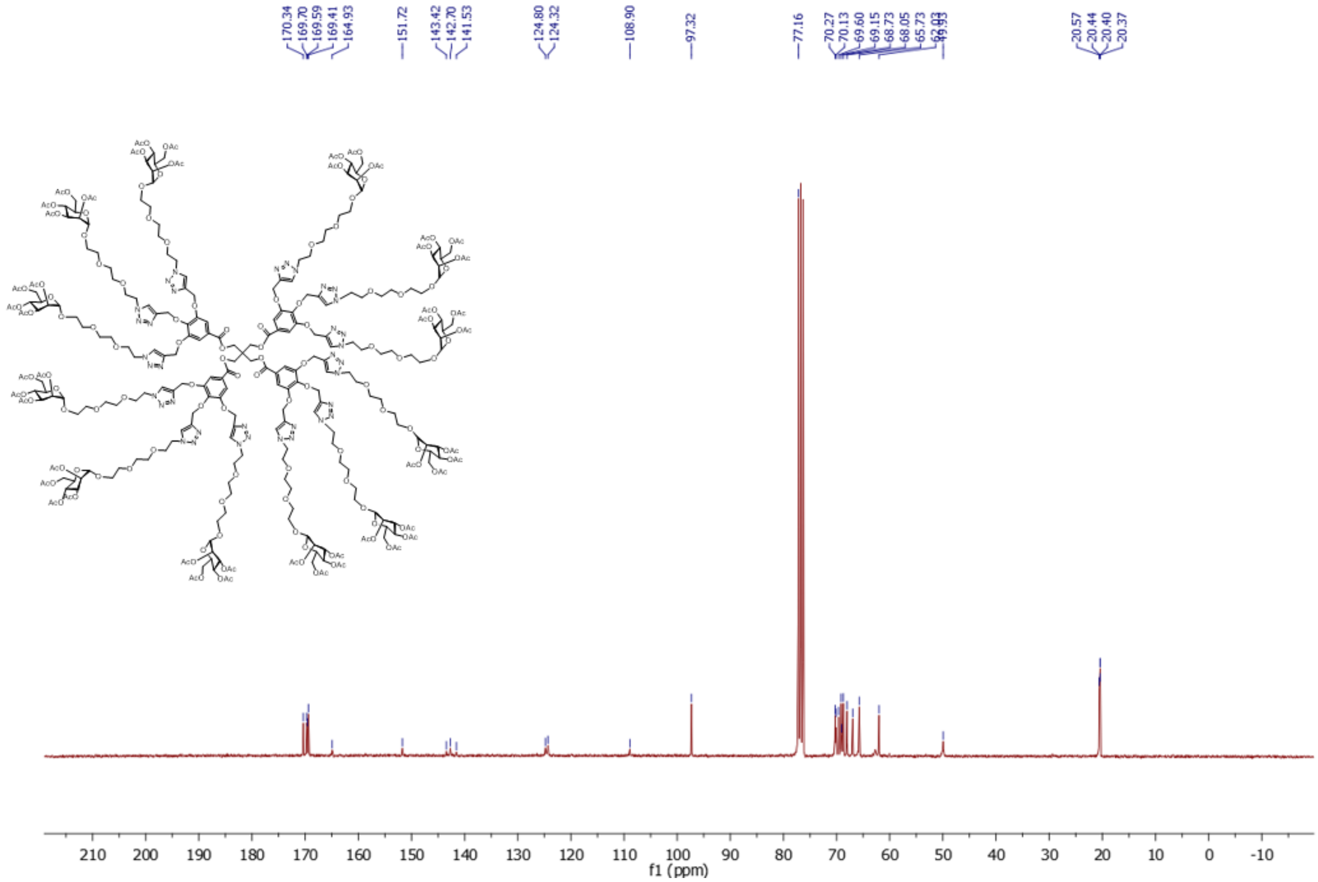

Figure S4. ^{13}C-NMR (75 MHz, $CDCl_3$) of compound 10

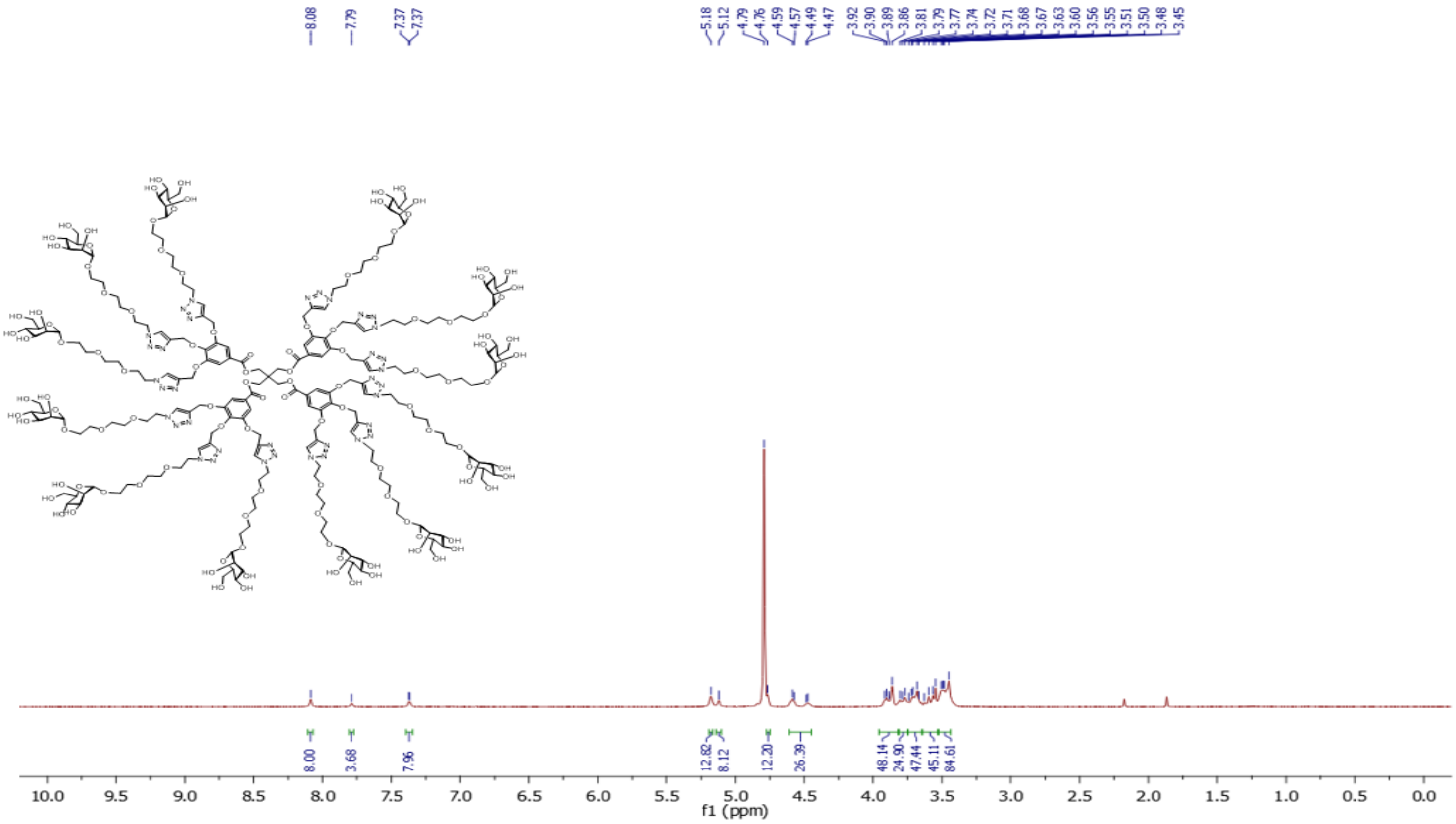

Figure S5. ^{1}H NMR (300 MHz, D_2O) of compound 11

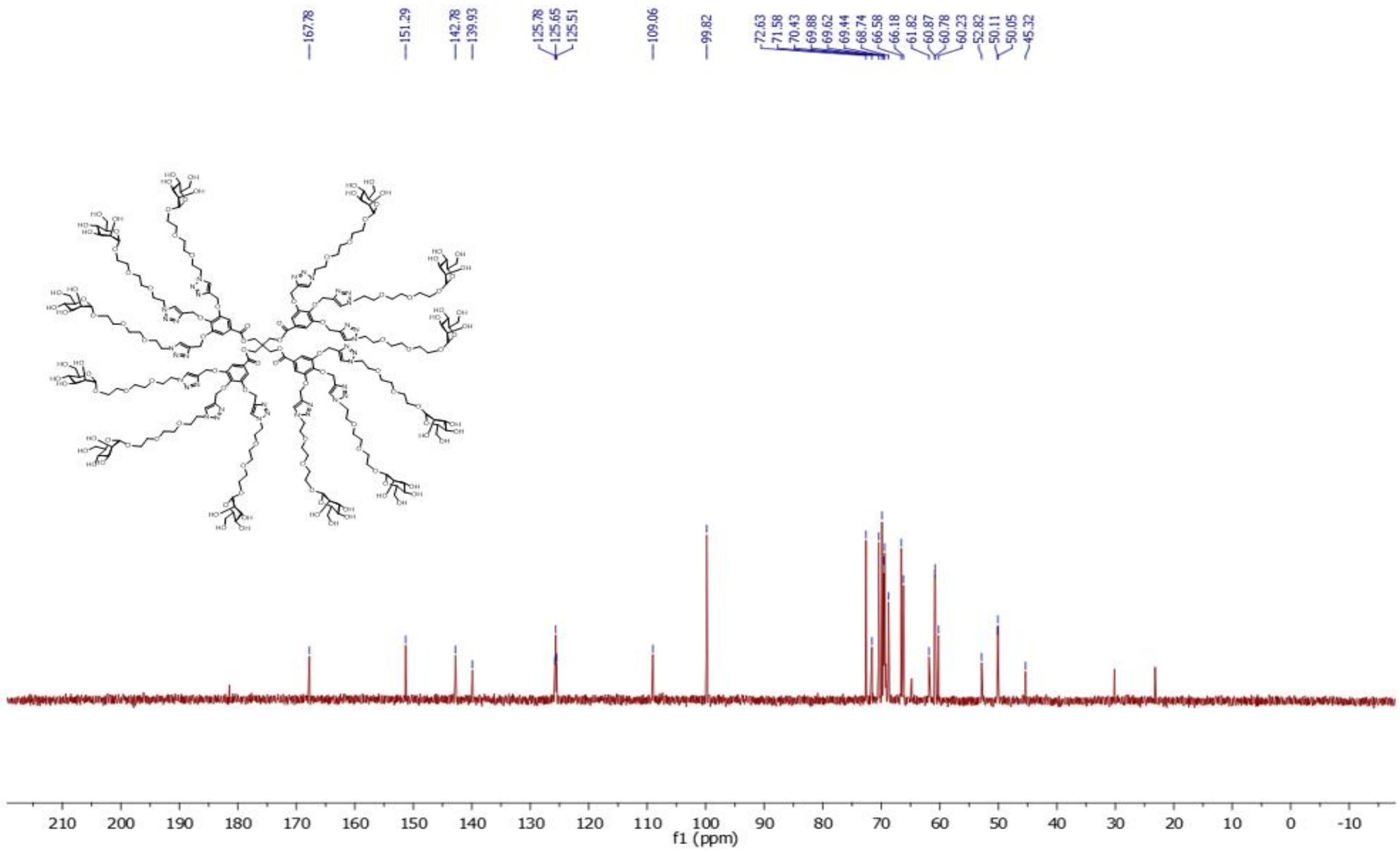

Figure S6. ^{13}C NMR (75 MHz, D_2O) of compound 11

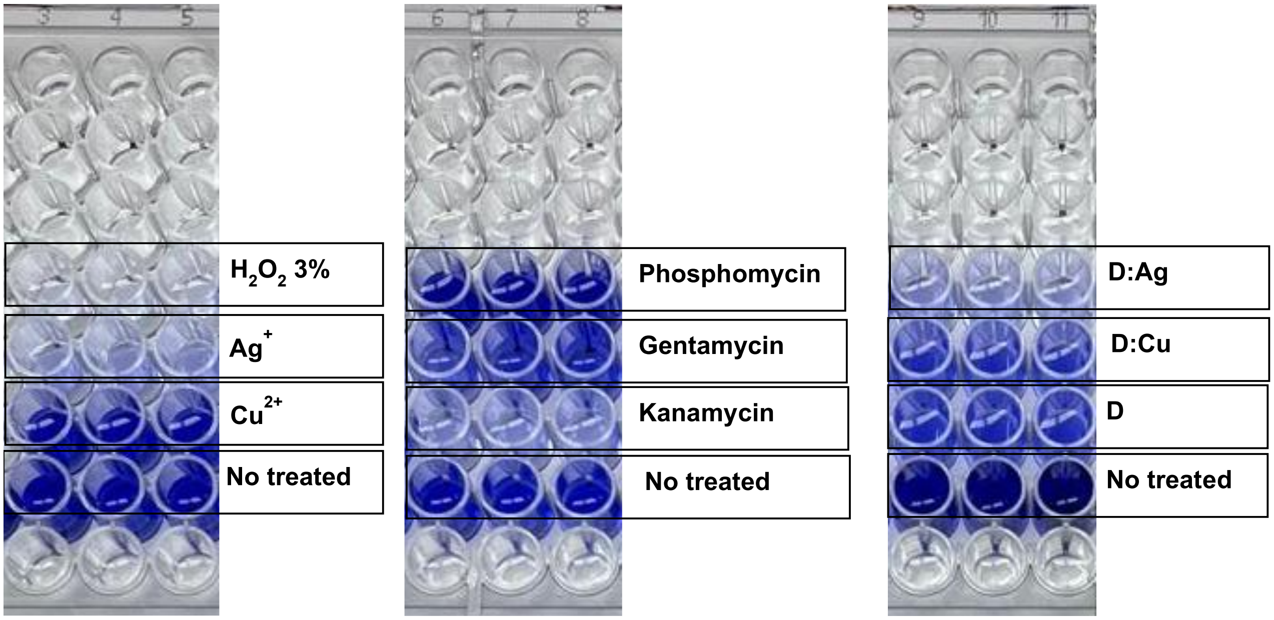

Figure S7. Biofilm production capabilities of *E. coli* enterotoxigenic fimbriae 4 (ETEC:F4) in the absence or presence of bactericidal agents by crystal violet Assay.

Table S1. Antibiotics effect on ETEC:F4

Materials		Inhibition zone (cm)
Dendrimers	D	1.10 ± 0.14
	D:Cu	1.11 ± 0.15
	D:Ag	2.25 ± 0.25
Current used bactericidal agents	Gentamycin	1.02 ± 0.18
	Phosphomycin	1.04 ± 0.18
	Kanamycin	2.18 ± 0.19
Metal ions	Cu^{2+}	1.35 ± 0.12
	Ag^+	1.35 ± 0.12
Components of dendrimer synthesis	Gallic acid	0.78 ± 0.05
	mannose	0.98 ± 0.13
Positive control	H_2O_2	3.15 ± 0.21

Average diffusion diameters as growth inhibition of bacteria induced by 1 mg of dendrimeric materials, of current antibacterial agents usually used and of 10 μL of 3% H_2O_2
